# A New Approach for the AFM-Based Mechanical Characterization of Biological Samples

**DOI:** 10.1155/2020/2896792

**Published:** 2020-10-18

**Authors:** S. V. Kontomaris, A. Malamou, A. Stylianou

**Affiliations:** ^1^Athens Metropolitan College, Faculty of Architecture, Engineering and Built Environment, Athens, Greece; ^2^Radar Systems and Remote Sensing Lab of School of Electrical & Computer Engineering of National Technical University of Athens, Greece; ^3^Cancer Biophysics Laboratory, Department of Mechanical and Manufacturing Engineering, Faculty of Engineering, University of Cyprus, Cyprus; ^4^School of Science, European University Cyprus, Cyprus

## Abstract

The AFM nanoindentation technique is a powerful tool for the mechanical characterization of biological samples at the nanoscale. The data analysis of the experimentally obtained results is usually performed using the Hertzian contact mechanics. However, the aforementioned theory can be applied only in cases that the sample is homogeneous and isotropic and presents a linear elastic response. However, biological samples often present depth-dependent mechanical properties, and the Hertzian analysis cannot be used. Thus, in this paper, a different approach is presented, based on a new physical quantity used for the determination of the mechanical properties at the nanoscale. The aforementioned physical quantity is the work done by the indenter per unit volume. The advantages of the presented analysis are significant since the abovementioned magnitude can be used to examine if a sample can be approximated to an elastic half-space. If this approximation is valid, then the new proposed method enables the accurate calculation of Young's modulus. Additionally, it can be used to explore the mechanical properties of samples that are characterized by a depth-dependent mechanical behavior. In conclusion, the proposed analysis presents an accurate yet simple technique for the determination of the mechanical properties of biological samples at the nanoscale that can be also used beyond the Hertzian limit.

## 1. Introduction

The most extensively used method for the determination of the mechanical properties of biological samples at the nanoscale is the AFM nanoindentation method. This method is based on the application of a specific load to a nanoregion and the subsequent measurement of the indentation into the sample [1]. The load-indentation data is then fitted to basic models of applied mechanics, and Young's modulus can be easily calculated as a fitting parameter (under the condition that Poisson's ratio of the sample and the indenter's properties are known) [2]. Using this simple approach, Young's modulus maps of extended nanoregions can be created for various applications [3–6]. The data processing is usually performed using basic models from contact mechanics like the Hertzian analysis [7]. The Hertz model has been proven to be a powerful tool for a wide range of applications [8–12]. However, it can be applied only under specific restrictions; the sample is considered as homogeneous and isotropic and presents a linear elastic response [7, 13]. This assumption has been proven rational in many cases in the literature [14, 15]. However, for big indentation depths and for highly nonhomogeneous and nonisotropic samples, Young's modulus calculation using the Hertzian analysis provides significant errors [7]. It must be also noted that these errors are usually “hidden” since the related software packages often present Young's modulus maps of large regions (e.g., using thousands of load-indentation curves) based on significantly inaccurate fittings [16, 17]. In addition, in the provided Young's modulus maps, a combination of accurate and inaccurate fittings (depending on the nanoregion) is included, and as a result, the calculation of Young's modulus distribution which is a basic tool for the evaluation of the mechanical properties of biological samples (e.g., for the detection of various pathological conditions [4, 18]) can be inaccurate.

Thus, according to the abovementioned facts, there are two significant objectives regarding nanoindentation experiments. The first objective is to find an accurate method which can be used as a tool to examine if the load-indentation data follows the Hertzian mechanics. The second objective is to provide a simple method to evaluate the mechanical properties of biological samples beyond the Hertzian limit (provided that the sample can be considered as a half-space, i.e., the sample's dimensions are significantly bigger compared to the tip's dimensions). Hence, in this paper, we introduce a new physical quantity that can be used for data processing in AFM nanoindentation experiments, the work done by the indenter per unit volume.

## 2. Materials and Methods

### 2.1. Open Access Data and Software Analysis

For the purposes of this paper, open access data obtained from nanoindentation experiments on fibroblasts was used [19]. In particular, over 130 loading load-indentation curves were analyzed. According to the research group that conducted the experiments, a conical indenter with a half angle equal to 25° was employed. In addition, the cantilever's spring constant was measured 0.01 N/m [19]. The load-indentation curves (using the raw load-displacement data collected from AFM) were constructed using the AtomicJ software and the protocol presented in [19]. The contact point determination and the fitting procedures according to Hertzian contact mechanics were also performed using the AtomicJ software [19]. Calculations of the work done by the indenter per unit volume were performed using Matlab.

### 2.2. The Work Done per Unit Volume as a Physical Quantity

In an AFM nanoindentation experiment, the applied load is related to the indentation depth using the following general equation [2],

(1) $$\begin{array}{c} P=ah^{m}. \end{array}$$

In equation (1), *P* is the applied load, *h* is the indentation depth, and *a*, *m* are constants that depend on the shape of the indenter and on the material's properties [2, 17]. The work done by the indenter can be easily calculated as follows [20]:

(2) $$\begin{array}{c} W=\int_{0}^{h_{\max}}Pdh=\frac{a}{m+1}h_{\max}^{m+1}. \end{array}$$

In addition, the contact stiffness of the material can be easily derived [21–23]:

(3) $$\begin{array}{c} S={\left.\frac{dP}{dh}\right|}_{h_{\max}}=amh_{\max}^{m-1}. \end{array}$$

According to the general equation that is valid for any axisymmetric indenter when indenting an elastic half-space, the ratio of the contact stiffness (*S*) of the material with respect to the contact radius (*r*_*C*_) is constant [24]:

(4) $$\begin{array}{c} \frac{S}{r_{c}}=\frac{2E}{1-v^{2}}. \end{array}$$

In equation (4), *E*, *v* are the sample's Young's modulus and Poisson's ratio, respectively. The combination of equations (3) and (4) results in

(5) $$\begin{array}{c} r_{c}=\frac{am\left(1-v^{2}\right)}{2E}h_{\max}^{m-1}=ch_{c}^{m-1}, \end{array}$$

where *c* = *am*(1 − *v*^2^)*b*^*m*−1^/2*E* and *b* = *h*_max_/*h*_*c*_, where *h*_*c*_ is the contact depth [21].

In addition, the volume of the part of the indenter which is at contact with the elastic half-space can be easily calculated:

(6) $$\begin{array}{c} V=\int_{0}^{h_{c}}\pi r^{2}dh=\int_{0}^{h_{c}}\pi c^{2}h^{2\left(m-1\right)}dh=\frac{\pi c^{2}}{2m-1}h_{c}^{2m-1}. \end{array}$$

The work done by the indenter per unit volume in the elastic material is provided as follows:

(7) $$\begin{array}{c} \frac{W}{V}=\frac{\left(a/\left(m+1\right)\right)h_{\max}^{m+1}}{\left(\pi c^{2}/\left(2m-1\right)\right)h_{c}^{2m-1}}=\frac{a\left(2m-1\right)b^{2m-1}}{\pi c^{2}\left(m+1\right)}h_{\max}^{2-m}. \end{array}$$

### 2.3. Conical Indenters and Young's Modulus Calculation

The ratio *W*/*V* is constant (i.e., it does not depend on the maximum indentation depth) if *m* = 2. The case of *m* = 2 is the case of conical indentation, where *a* = (2/*π*)(*E*/(1 − *v*^2^))tan*θ*, *b* = *π*/2, and *θ* is the cone's half angle [2, 22]. Thus, in this case, equation (7) can be written in the form

(8) $$\begin{array}{c} \frac{W}{V}=\frac{\pi E}{4\left(1-v^{2}\right)\tan\theta }=\text{const}. \end{array}$$

**Figure 1:**
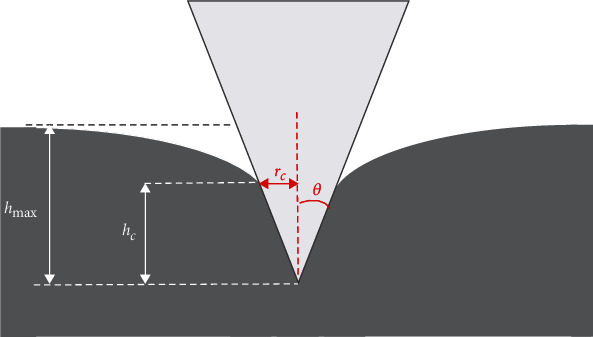
An illustration of a nanoindentation experiment using a conical indenter on an elastic half-space. The contact depth (*h*_*c*_) and the contact radius (*r*_*c*_) are clearly presented.

Thus, if the ratio *W*/*V* is constant for a specific sample and for different indentation depths (using a conical indenter), this sample can be considered as homogeneous and isotropic and Young's modulus can be easily determined using equation (8). An illustration of a nanoindentation experiment using a conical indenter on an elastic half-space is presented in Figure 1. For nonconical indenters, the ratio *W*/*V* is depth-dependent for any sample and for every indentation depth. For example, assume a spherical indenter. In this case, *m* = 3/2, *a* = (4/3)(*E*/(1 − *v*^2^))*R*^1/2^, and *b* = 2 [1, 22], and as a result, even when the sample can be approximated to an elastic half-space, the ratio *W*/*V* is not constant and follows the equation

(9) $$\begin{array}{c} \frac{W}{V}=\frac{32}{15}\frac{E}{1-v^{2}}R^{-1/2}h_{\max}^{1/2}. \end{array}$$

As a result, the presented analysis can be applied more easily if the indenter can be approximated to a perfect cone. It is also interesting to mention that the units of the proposed physical quantity *W*/*V* are equivalent to Pascal's (i.e., J/m^3^ = N/m^2^ = Pa).

### 2.4. Testing the Mechanical Behavior of a Sample

An easy way to test if the ratio *W*/*V* is constant (using a conical indenter) is to plot the graph *W* = *f*(*h*^3^), since the volume of the indenter which is in contact with the sample increases proportionally to *h*^3^. The work done by the indenter for each indentation depth (0 ≤ *h* ≤ *h*_max_) can be easily calculated from the area under the *P* = *f*(*h*) graph. If the graph *W* = *f*(*h*^3^) is linear, the ratio *W*/*V* is constant and the sample can be accurately described by Young's modulus as provided by Hertzian analysis.

On the other hand, if the aforementioned ratio is not constant, equations (4), (5), (7), and (8) are no longer valid. In other words, the sample cannot be considered as an elastic half-space. In this case, the ratio *W*/*V* is depth-dependent:

(10) $$\begin{array}{c} \frac{W}{V}=f\left(h\right). \end{array}$$

Thus, by calculating the *W*/*V* ratio with respect to the indentation depth, the depth dependence of the sample's mechanical properties can be accurately determined. The volume which is in contact with the sample can be easily calculated using equation (6) for a conical indenter (*m* = 2, *h*_*c*_ = (2/*π*)*h*_max_, and *a* = (2/*π*)(*E*/(1 − *v*^2^))tan*θ*):

(11) $$\begin{array}{c} V=\frac{8\text{ta}{\text{n}}^{2}\theta }{3\pi^{2}}h_{\max}^{3}. \end{array}$$

## 3. Results and Discussion

### 3.1. Data Processing

**Figure 2:**
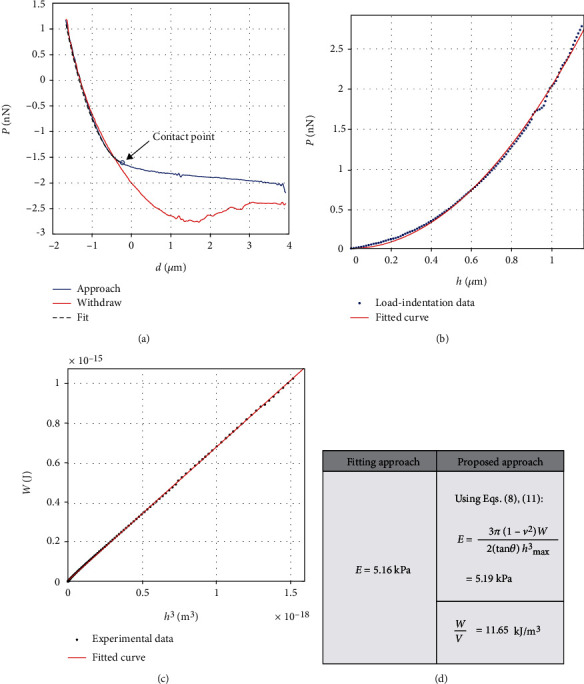
(a) AFM load-displacement curves, (b) loading load-indentation (*P* = *f*(*h*)) data and a fit to equation (12) (*R*^2^ = 0.9984), (c) the *W* = *f*(*h*^3^) data present a linear behavior (*R*^2^ = 0.9998), and (d) Young's modulus as calculated using a fit to equation (12) and using the proposed by this paper approach. The difference is negligible, since the sample presents a linear elastic behavior for the selected data range. The *W*/*V* ratio is also presented.

In Figure 2, a load-displacement curve (Figure 2(a)) and the resulting loading load-indentation curve (Figure 2(b)) obtained on a fibroblast are presented. The loading load-indentation data fits well to the equation provided by Sneddon (which is an extension of Hertzian analysis) for conical indenters [2]:

(12) $$\begin{array}{c} P=\frac{2}{\pi }\frac{E}{\left(1-v^{2}\right)}\left(\tan\theta \right)h^{2}. \end{array}$$

In particular, the fitted curve is described by the function, *P* = (2042(N/m^2^))*h*^2^, 0 ≤ *h* ≤ 1149 nm(*R*^2^ = 0.9984). Assuming Poisson's ratio equal to *v* = 0.5, Young's modulus results in *E* = 5.16 kPa. However, the aforementioned value is valid only under the condition that the ratio *W*/*V*  remains constant at the domain 0 ≤ *h* ≤ 1149 nm. Thus, in Figure 2(c), the graph *W* = *f*(*h*^3^) is presented which is linear (*R*^2^ = 0.9998). As a result, the ratio *W*/*V* is constant and the sample can be accurately described by Hertzian mechanics.

Young's modulus was also calculated using the proposed by this paper analysis. For this reason, the area under the experimental data (Figure 2(a)) which equals the work done by the indenter was used and resulted equal to *W* = 10.37 · 10^−7^ nJ. The volume of the indenter which is in contact with the sample was calculated *V* = 8.9 · 10^7^ nm^3^ (equation (11)). Thus, *W*/*V* = 11.65 kJ/m^3^. Hence, using equation (8), Young's modulus results in 5.19 kPa. The differences between the two approaches are negligible.

**Figure 3:**
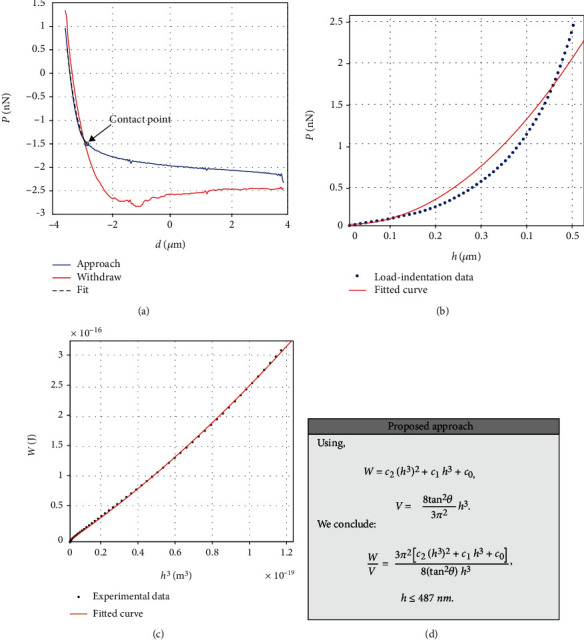
(a) AFM load-displacement curves, (b) loading load-indentation (*P* = *f*(*h*)) data and a fit to equation (12). In this case, the fitting is poor (*R*^2^ = 0.9591). (c) The *W* = *f*(*h*^3^) data present a depth-dependent behavior since it is fitted to a second-degree polynomial curve (*R*^2^ = 0.9997). (d) The related equations for the calculation of *W*/*V* ratio at the indentation depth of interest.

In Figures 3(a) and 3(b), the load-displacement curves and the resulting loading load-indentation curve on a different point (on the same fibroblast) are presented. In this case, the fitting of the data to equation (12) is poor (*R*^2^ = 0.9591). As it was expected, the graph *W* = *f*(*h*^3^) is not linear in this case (Figure 3(b)). Thus, the mechanical properties at this nanoregion cannot be described accurately using Young's modulus as provided by Hertzian contact mechanics. In other words, the mechanical properties at this nanoregion present a depth-dependent behavior. The *W* = *f*(*h*^3^) data was fitted to a 2^nd^-degree polynomial curve:

(13) $$\begin{array}{c} y=c_{2}x^{2}+c_{1}x+c_{0}. \end{array}$$

In equation (13), *c*_2_ = 5 · 10^21^ J/m^6^,  *c*_1_ = 2014 J/m^3^, *c*_0_ = 3.548 · 10^−18^ J, *x* = *h*^3^, 0 ≤ *h* ≤ 487 nm, and *R*^2^ = 0.9997. For example, according to equations (10), (11), and (13), if

(14) $$\begin{array}{c} h=380\text{ nm},\frac{W}{V}=39.84\text{ kJ}/{\text{m}}^{3},\\ h=430\text{ nm},\frac{W}{V}=41.30\text{ kJ}/{\text{m}}^{3},\\ h=487\text{ nm},\frac{W}{V}=44.79\text{ kJ}/{\text{m}}^{3}. \end{array}$$

The depth-dependent behavior is probably a result of the nonhomogeneity of the sample or a result of a substrate effect [25]. However, regardless of the reason for this behavior, it was shown that the ratio *W*/*V* can be used as a physical quantity to describe the behavior of a sample not only for those that can be considered homogeneous but also for those beyond the Hertzian limit.

It must be noted that the proposed by this paper analysis assumes a perfect conical indenter. However, the analysis can be used for tips with a *n*-sided pyramid shape as well (*n*-sided pyramid shape tips are often used in AFM nanoindentation experiments).

### 3.2. Mechanical Property Maps and Statistics

**Figure 4:**
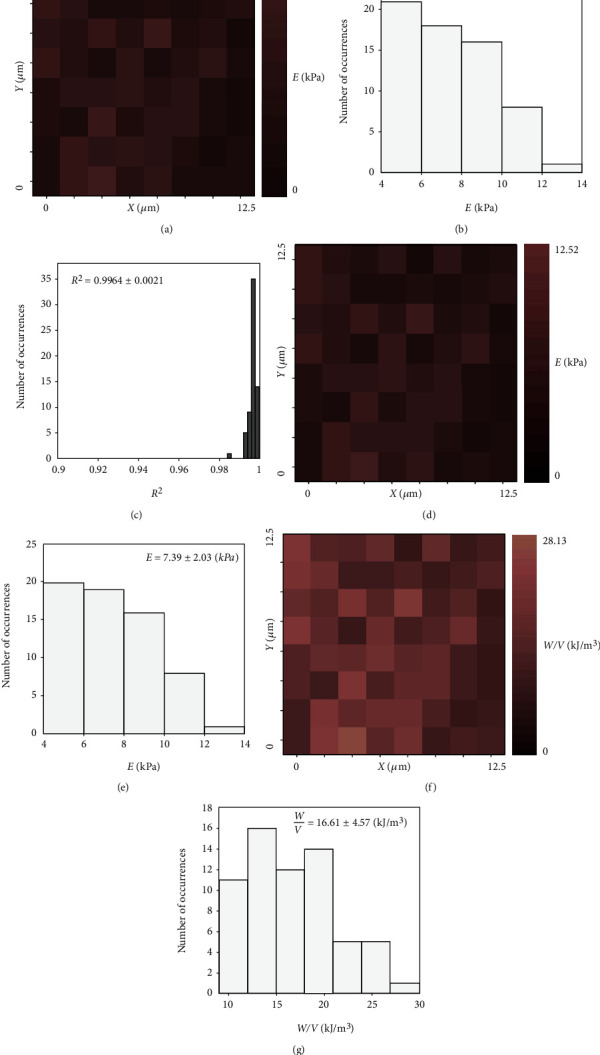
(a) Young's modulus map consisting of 64 measurements over a region 12.5 *μ*m × 12.5 *μ*m. (b) The distribution of Young's modulus values. The mean ± standard deviation resulted in *E* = 7.41 ± 2.00 (kPa). (c) The data was accurately fitted to equation (12) since *R*^2^ for each individual fit was close to 1. (d) Young's modulus map over the same region using the proposed by this paper approach. (e) Young's modulus distribution reveals that the new approach can be accurately used for Young's modulus calculation under the condition that the sample presents a linear elastic behavior. (f) A mechanical property map in terms of *W*/*V*. (g) The distribution of the *W*/*V* values. The mean ± standard deviation resulted in 16.61 ± 4.57 kJ/m^3^.

In this section, measurements over extended regions on a fibroblast will be presented. In particular, in Figure 4, the analysis of 64 loading load-indentation curves is presented (over a region 12.5 *μ*m × 12.5 *μ*m, at the central region of a fibroblast). The maximum indentation depth for each curve was chosen to be ~1000 nm.

Firstly, the data was fitted to equation (12), and the *R*-squared coefficient (mean ± standard deviation) resulted in *R*^2^ = 0.9964 ± 0.0021. Hence, the data follows Sneddon's equation in this case. As a result, Young's modulus map using the aforementioned fitting procedure is created (Figure 4(a)). In addition, the distribution of Young's modulus values is presented in Figure 4(b). The mean ± standard deviation regarding Young's modulus values resulted in *E* = 7.41 ± 2.00 (kPa). In Figure 4(c), the distribution of *R*^2^ is also presented (in every case, *R*^2^ was close to 1).

Furthermore, Young's modulus for each curve was calculated using the alternating procedure proposed by this paper (equation (8)). The results are presented in Figures 4(d) and 4(e). Young's modulus map (Figure 4(d)) is identical to Young's modulus map in Figure 4(a). In addition, the mean ± standard deviation of Young's modulus value resulted in *E* = 7.39 ± 2.03 (kPa).

Using the above-mentioned results, it can be concluded that if the sample presents a linear elastic behavior (*W*/*V* = const.), then the approach proposed by this paper can be used as an alternating method regarding Young's modulus calculations (the fact that Young's modulus distribution resulted identical using the two methods is also a proof that the sample's behavior was consistent with a linear elastic response over the selected range of indentation depths). Additionally, the mechanical property map in terms of *W*/*V* is also presented. The mean ± standard deviation of *W*/*V* value resulted in 16.61 ± 4.57 kJ/m^3^.

**Figure 5:**
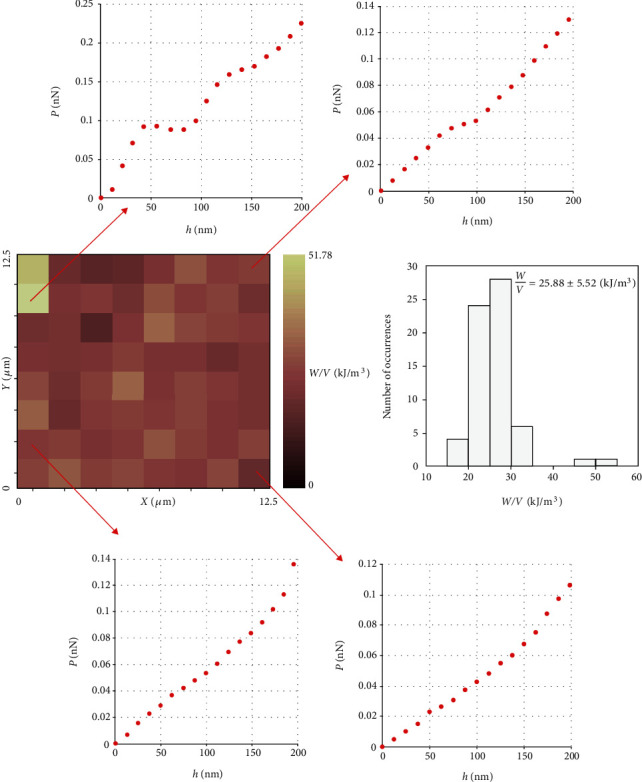
If a sample does not present a linear elastic behavior, a mechanical property map in terms of *W*/*V* can be used. The aforementioned map is presented, consisting of 64 measurements. The range of data that were analyzed in this case was the data that represents the first 200 nm of the indentation. Four randomly selected loading load-indentation curves are presented to show that a fitting to Sneddon's equation is not an appropriate solution in this case. The *W*/*V* distribution is also presented. The mean ± standard deviation resulted in 25.88 ± 5.52 (kJ/m^3^).

Furthermore, an additional mechanical property map on a different location of the fibroblast is presented in Figure 5. In this case, only the data that represents the first 200 nm of the indentation was analyzed. Fitting equation (12) to the data and determining Young's modulus using Hertzian analysis were not an appropriate method to estimate the mechanical properties since the *R*-squared coefficient resulted in the range 0.7576 ≤ *R*^2^ ≤ 0.9872 (the mean ± standard deviation of the *R*-squared coefficient resulted in *R*^2^ = 0.9455 ± 0.0479). This is an expected result, since previous publications have shown that the mechanical properties of cells are usually highly depth-dependent for small indentation depths [26–28] (it must be noted that using the analysis conducted by this paper, it was concluded that most of the fibroblast's central regions had a linear elastic behavior if the maximum indentation depth was approximately in the range 400 nm ≤ *h*_max_ ≤ 1000 nm). Thus, in this case, the determination of Young's modulus using equation (12) is an inappropriate method to estimate the sample's mechanical properties. In this case, a mechanical property map in terms of *W*/*V* is the most appropriate solution. More specifically, a *W*/*V* map consisting of 64 measurements over a region 12.5 *μ*m × 12.5 *μ*m is presented in Figure 5. Four randomly selected load-indentation curves are also shown to prove that a fitting using equation (12) to the data is meaningless in this case. The mean ± standard deviation of the *W*/*V* magnitude resulted in 25.88 ± 5.52 (kJ/m^3^). The *W*/*V* distribution is also presented using a histogram in Figure 5.

### 3.3. Advantages of the New Method

In this paper, the physical magnitude “work per unit volume” is introduced as an accurate physical quantity for the mechanical characterization of biological samples at the nanoscale using a perfect conical indenter. The presented by this paper analysis depicts significant advantages since

- It can be used as a test to evaluate whether the Hertzian contact mechanics (equation (12)) can be used for Young's modulus determination at the nanoscale
- It provides an alternating method for Young's modulus determination under the condition that *W*/*V* = const.
- It can be used to explore the mechanical properties of samples which present a depth-dependent behavior, and as a result, the Hertzian analysis is of no use
- It can be used for the comparison of the mechanical properties of different samples beyond the linear elastic regime
- The *W*/*V* ratio can be used to explore the depth at which the sample exceeds the linear elastic behavior
- The *W*/*V* calculations can be included in basic software packages regarding data processing in AFM nanoindentation experiments to exclude measurements that do not present constant *W*/*V* ratios. As a result, the final Young's modulus distributions will be accurate and likely to be used in the future in real clinical activities
- It provides a simple magnitude in terms of J/m^3^, with physical significance which is (like Young's modulus) easily to be understood by professionals in the fields of biology and medicine. For example, the comparison of materials with depth-dependent mechanical properties using the same indentation depth is easy; the stiffer material requires “more work” to be indented using the same indenter
- The advantage of not needing a fit allows faster determination of Young's modulus and, thus, opens the possibility of real-time analysis
- The *W*/*V* ratio can be also used to determine the maximum indentation depth that should not be surpassed in order to avoid the substrate effects in cases that linear elastic samples are being tested. In particular, if a linear elastic sample is being tested, the *W*/*V* ratio is constant and the *W* = *f*(*h*^3^) curve is linear. However, after a limit, the *W* = *f*(*h*^3^) curve will exceed the linear form due to the substrate effect. Thus, only the first linear data (prior to the above-mentioned limit) should be used to determine the sample's mechanical properties. The substrate's influence on Young's modulus measurements is a classic problem in AFM nanoindentation experiments. Significant theories have been also proposed in the past for data processing to render the mechanical properties of linear elastic or viscoelastic samples regardless of the value of the maximum indentation depth [29–31]. Thus, this paper is also a new contribution in this direction

### 3.4. Limitations

It must be noted that the proposed approach has also some limitations which are provided below:

- It must be strictly clarified that the analysis as provided by this paper assumes a perfect conical approximation of the indenter. However, this is usually the case in AFM nanoindentation experiments since most of the indenters have pyramidal shapes and can be approximated to perfect cones for big nanoindentation depths (compared to the tip apex radius)
- It must be also noted that the comparison of the mechanical properties of different samples that present depth-dependent behavior using the ratio *W*/*V* can be performed only for the same indentation depth and when using the same indenter type (same cone's half angle)
- By using the proposed method by this paper, it is not possible to conclude if a depth-dependent mechanical behavior is a result of the sample's nonhomogeneity or due to a substrate effect (in the case that it is not known if the tested sample should present or not a linear elastic behavior). Thus, special attention should be given to Buckle's rule [25]. In particular, the maximum indentation depth cannot exceed the 5–10% of the sample's thickness

### 3.5. An Analogy with Electrical Components

The examination of the *W*/*V* values as presented in this paper for a biological sample can be considered as an analogy to the examination of the ratio *V*′/*I* for an electric component, where *V*′ is the voltage difference between the terminals of the component and *I* the current through it. More specifically, if the ratio *V*′/*I* is constant, then the component presents a linear behavior (e.g., resistor). In this case, the component can be described by Ohm's law since the resistance of the component is constant (*R* = const.). Otherwise, the ratio *V*′/*I* can be also used for the characterization of the behavior of the component by using the function *I* = *f*(*V*′) (e.g., diode and transistor).

## 4. Conclusion

In this paper, a new methodology for data processing in AFM nanoindentation experiments was presented. Using the proposed analysis, it can be easily evaluated whether the Hertzian analysis is the correct approach for data processing. In addition, the presented methodology provides significant advantages regarding the investigation of samples that present depth-dependent mechanical properties. Thus, it is a valuable tool that can be used in combination with the basic Hertzian theory to provide significant additional information regarding the mechanical behavior of a sample.

## Data Availability

The (load-indentation) data used to support the findings of this study have been deposited in the (AtomicJ) repository (https://sourceforge.net/projects/jrobust/files/TestFiles/).

## Additional Points

*Future Work*. The presented analysis will be used to explore the depth dependence of the mechanical properties of normal, benign, and cancerous tissues.
